# Genetic Code Expansion: A Powerful Tool for Understanding the Physiological Consequences of Oxidative Stress Protein Modifications

**DOI:** 10.1155/2018/7607463

**Published:** 2018-04-23

**Authors:** Joseph J. Porter, Ryan A. Mehl

**Affiliations:** Department of Biochemistry and Biophysics, Oregon State University, 2011 Agriculture and Life Sciences Building, Corvallis, OR 97331, USA

## Abstract

Posttranslational modifications resulting from oxidation of proteins (Ox-PTMs) are present intracellularly under conditions of oxidative stress as well as basal conditions. In the past, these modifications were thought to be generic protein damage, but it has become increasingly clear that Ox-PTMs can have specific physiological effects. It is an arduous task to distinguish between the two cases, as multiple Ox-PTMs occur simultaneously on the same protein, convoluting analysis. Genetic code expansion (GCE) has emerged as a powerful tool to overcome this challenge as it allows for the site-specific incorporation of an Ox-PTM into translated protein. The resulting homogeneously modified protein products can then be rigorously characterized for the effects of individual Ox-PTMs. We outline the strengths and weaknesses of GCE as they relate to the field of oxidative stress and Ox-PTMs. An overview of the Ox-PTMs that have been genetically encoded and applications of GCE to the study of Ox-PTMs, including antibody validation and therapeutic development, is described.

## 1. Introduction

It is accepted that posttranslational modifications resulting from oxidation (Ox-PTMs) damage proteins and harm cells. Whether Ox-PTMs can modulate the function of proteins in a specific manner like other PTMs has been a long-standing question [1]. Recent studies have demonstrated that site-specific protein Ox-PTMs can lead to notable gain-of-function alterations that are connected to disease phenotypes. Enzymatic pathways that remove Ox-PTMs have also been identified, providing evidence for dynamic homeostasis with implications for the cellular function of these modifications. Major challenges exist in evaluating effects of Ox-PTMs because of the diversity of mechanisms by which they are formed. The Ox-PTMs result from reactive oxygen species (ROS) (superoxide (O_2_^•−^), hydrogen peroxide (H_2_O_2_), and hydroxyl radical (OH^•^)), reactive nitrogen and oxygen species (RNS) (nitric oxide (^•^NO), nitrogen dioxide (^•^NO_2_), and peroxynitrite (ONOO^−^/ONOOH)), and other downstream products interacting with a variety of amino acid residues [2–8].

**Figure 1:**
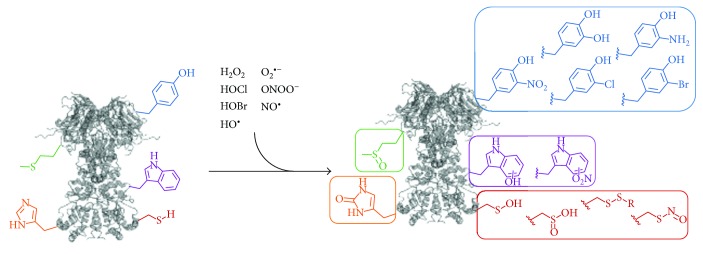
A variety of biological oxidants are capable of modifying susceptible amino acid sidechains to their Ox-PTM counterparts. The major groups of amino acids modified are the sulfur containing amino acids (cysteine and methionine) and the aromatic amino acids (tyrosine, tryptophan, and histidine).

Elucidating the function of PTMs is notoriously challenging to evaluate because generating the modified proteins *in vivo* or *in vitro* results in a heterogeneous mixture of modified and unmodified proteins. The situation for oxidative stress PTMs is notably more dire because the installation method, diverse and nonspecific chemical reactions from ROS and RNS, produces a heterogeneous mixture of Ox-PTMs on proteins containing multiple different modifications (Figure 1). Each different Ox-PTM needs to be assessed for site-specificity and abundance to identify its effects on protein function. Genetic code expansion (GCE) is particularly well suited to meet these challenges since its core GCE cotranslationally installs the Ox-PTM as a noncanonical amino acid (ncAA). This allows for facile production of homogenously modified protein at genetically programed sites, enabling new approaches for studying Ox-PTMs. GCE can validate Ox-PTM residues identified in oxidative stress conditions and explore the functional consequences of a single site of modification. GCE can also be used to develop assays for a particular site of modification on a particular protein and to generate controls for evaluating the selectivity and effectiveness of antibodies for Ox-PTMs. Since GCE functions by generating ncAA-protein in living prokaryotes and eukaryotes, it also allows for the *in vivo* study of homogeneous site-specifically modified protein.

**Figure 2:**
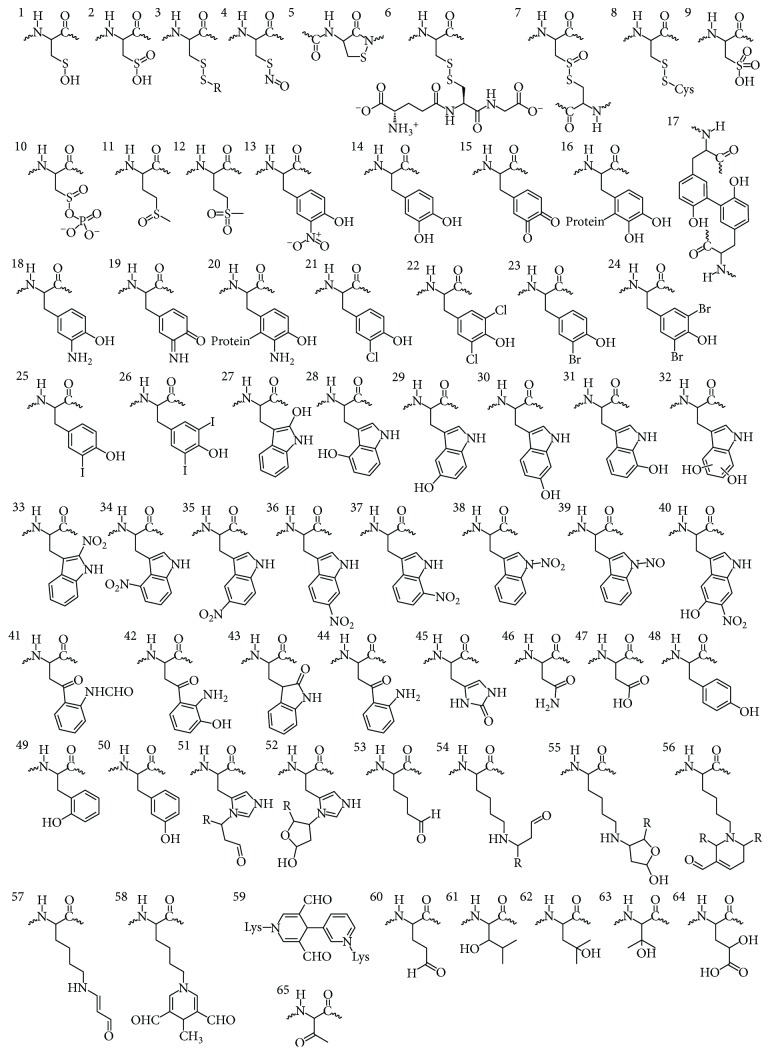
Ox-PTMs identified on proteins isolated from conditions of oxidative stress or following *in vitro* reaction with ROS or RNS. For the list of Ox-PTM names and references see the Supplementary Information Table 1.

A survey of the literature produces at a minimum 65 reported Ox-PTMs (Figure 2 and Supplementary Table 1). A number of excellent reviews on the ROS and RNS exist and as such we will not discuss them here [9, 10]. Identification of sites and identities of Ox-PTMs has also blossomed as a field of study, and these have been reviewed elsewhere [11, 12]. It is critical to note from studies on the identification of Ox-PTMs that these modifications are often not stable to purification from their native environments and require specialized stabilization or trapping methods [13]. This is important for GCE as these particularly sensitive Ox-PTMs will likewise require chemical caging strategies or stable mimetics for successful genetic incorporation. In this review, we summarize the relevant literature at the intersection of GCE and Ox-PTMs, focusing on the most abundant Ox-PTMs and those amenable to GCE technology (Figure 1). Ox-PTMs of low stability or amino acid cross-links which are not applicable to GCE will not be discussed (3, 7, 9-10, 16, 18–20, 22, 24–26, 31–35, 37–39, 41–44, 46–65 in Figure 2). This review will also highlight the strengths and shortcomings of GCE as applied to the study of Ox-PTMs, outline some of the important considerations when employing GCE, and describe exciting future applications of GCE technology for the oxidative stress field.

## 2. Genetic Code Expansion Orthogonal Systems

The ability to site-specifically incorporate noncanonical amino acids (ncAAs) into proteins in living cells has emerged as a powerful method to probe protein structure and function [14, 15]. This capability has been extended to the incorporation of many different PTMs including Ox-PTMs. While cell-free protein synthesis is also developing as a powerful approach for generating modified proteins [16], GCE is a technology that must work inside a living cell. A first consideration for GCE is that the Ox-PTM must be chemically synthesized as an amino acid, which can be challenging for some modifications. The modified amino acid must also be stable to cell culture conditions and be internalized in a cell to concentrations adequate for translation. The stability of an Ox-PTM to cell culture conditions should be evaluated because many Ox-PTMs are redox sensitive and can be toxic to cells at medium concentrations needed for GCE (0.1–1.0 mM). Provided that the Ox-PTM amino acid can pass these initial steps, then selection of GCE components specific for the new amino acid is possible.

**Figure 3:**
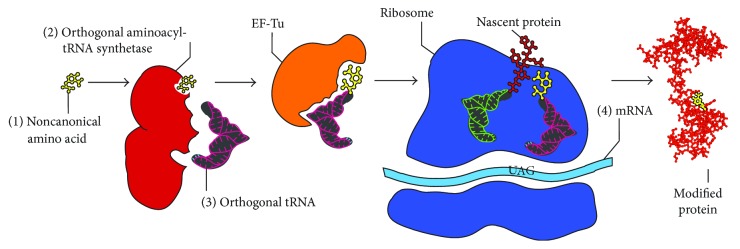
Components necessary for genetic code expansion including the noncanonical amino acid of interest (1), an orthogonal aminoacyl tRNA synthetase-tRNA pairs (2) and (3), and an mRNA with an amber stop codon at the site of interest (4).

Central to GCE technology is an engineered aminoacyl-tRNA synthetase-tRNA pair (aaRS-tRNA) that encodes an ncAA in response to a nonsense (often the amber stop codon) or a frameshift codon (Figure 3). To maintain translational fidelity the aaRS-tRNA pair must also not cross-react with any endogenous aaRS-tRNA pairs in the host organism, that is, this aaRS-tRNA pair must be orthogonal. In general, evolution of an orthogonal aaRS-tRNA pair in a cell requires importing this pair from another domain of life, as the aaRS-tRNA identity elements for recognition are divergent enough to maintain orthogonality. All of the aaRS-tRNA pairs employed so far for GCE were derived from an aaRS-tRNA pair for a canonical amino acid and were altered to instead recognize and charge an ncAA onto the orthogonal tRNA. Generally, this strategy has been more successful when the ncAA of interest resembles the original canonical amino acid (e.g., a modified TyrRS may only accept aromatic amino acids). As this trend exists, it is important to know what orthogonal systems have been used in the past when considering a heretofore unincorporated ncAA. Five main orthogonal pairs have been used, an archaeal TyrRS-tRNA_CUA_ pair from *Methanocaldococcus jannaschii (Mj)* has been used extensively in *E. coli* and other bacteria [17]; an *E. coli* LeuRS-tRNA_CUA_ and an *E. coli* TyrRS-tRNA_CUA_ pair have been used in eukaryotes [18, 19]; a pyrrolysyl-aaRS-tRNA pair (PylRS-tRNA_CUA_) derived from several methanogenic archaea (notably *Methanosarcina barkeri* and *Methanosarcina mazei*) is orthogonal in both *E. coli* and eukaryotes [20, 21]; and a liberated *E. coli* TrpRS-tRNA_CUA_ pair orthogonal in both *E. coli* and eukaryotes [22].

## 3. Developing Orthogonal Pairs

Initially, all orthogonal aaRS-tRNA pairs must be evolved to efficiently incorporate an ncAA of interest. A library of mutations at certain positions is constructed, and a double sieve selection scheme is employed to enrich aaRS variants that incorporate the ncAA of interest but not any of the canonical amino acids [17]. Following a successful selection, several parameters may be used to characterize the effectiveness of the developed aaRS-tRNA pair. The efficiency of the orthogonal pair is a measure of the amount of full length protein produced in the presence of ncAA and is often described by the fluorescence of a reporter protein like GFP or the yield of a purified protein of interest. Fidelity is a parameter that measures the orthogonality of the system. The absolute fidelity of an aaRS-tRNA pair is the amount of full length protein product produced in the absence of ncAA [23]. Relative fidelity is the amount of noncognate amino acid incorporated in the protein of interest in the presence of the ncAA of interest. Relative fidelity is a more useful parameter for characterization of an aaRS-tRNA pair as it is more closely resembles the fidelity of the aaRS under conditions in which it will be used. This parameter is measured by whole protein or tryptic digest mass spectrometry of the purified protein of interest to determine the amount of ncAA incorporation as compared to canonical amino acid. Oftentimes, an evolved orthogonal pair can incorporate a related family of ncAAs, for example, *para*-substituted phenylalanines [24]. This characteristic is called permissivity (sometimes referred to as polyspecificity) of an orthogonal pair [25].

## 4. Other Considerations and Alternatives to Genetic Code Expansion

There are factors beyond the aaRS/tRNA pair that are also critical for GCE. For a given ncAA to be incorporated by an evolved aaRS-tRNA pair, the ncAA has to meet a set of translational compatibility criteria. The bioavailability of the ncAA has to be taken into account as it must diffuse into, be transported into, or be synthesized within the cell. This is particularly an issue for highly charged amino acids which generally do not diffuse across membranes and a suitable endogenous transporter does not exist. Further, the ncAA of interest needs sufficient stability to persist intracellularly for a timescale on the order of hours to days in order to be incorporated into a protein via translation. Following aminoacylation of the tRNA by the aaRS, the EF-Tu must transport the aminoacyl-tRNA to the ribosome. The EF-Tu has been finely tuned for natural translation and, while it tolerates many ncAAs, those that are highly charged or particularly large are not effectively transported by the EF-Tu. The amino acyl-tRNA then must be decoded on the ribosome. Finally, the installed ncAA also needs to be stable on the protein enabling protein purification and characterization.

A variety of strategies have been employed to overcome these issues with GCE in regard to particular ncAAs. One solution to poor cellular uptake is conversion of the desired ncAA into a dipeptide. Dipeptides have been shown to increase uptake of highly charged or otherwise poorly internalized amino acids via transporters [26, 27]. Alternatively, methylation of the carboxylic acid of certain ncAAs also increases uptake in mammalian cell culture. A third solution is to generate a biosynthetic pathway for the desired ncAA so that it is generated inside the cell of interest [28, 29]. Generally, structural mimics or chemically caged derivatives of ncAAs are used in order to increase stability. For phosphorylated amino acids, both chemical caging [30] and structural mimetics [27, 31] have been used to stabilize the ncAA to allow for ncAA incorporation and characterization of PTM-proteins. Another strategy employed to increase the lifetime of genetically encoded PTM is to knock down the cell's PTM removal pathways. The lifetime of phosphoserine on proteins is increased by removal of endogenous serine phosphatases, allowing for genuine phosphoserine incorporation [31]. Generally, the EF-Tu transports ncAA aminoacylated-tRNAs efficiently enough to allow for incorporation but some charged ncAAs have required EF-Tu engineering. For initial studies on incorporation of phosphoserine, it was thought that it was necessary to evolve the EF-Tu to allow for the transport of phosphoserylated-tRNA; however, later studies indicated that while this evolved EF-Tu does transport phosphoserylated-tRNA more efficiently, evolution of the EF-Tu was not strictly necessary [31, 32]. The ribosome needs to accommodate the ncAA-tRNA and catalyze peptide chain formation. Chemically acylated tRNA and cell-free synthesis have confirmed that the ribosome is very permissive and ncAAs > 700 Da in size have been incorporated without issue [16]. While an ncAA size limit has been identified for the ribosome exit tunnel using cell-free protein synthesis methods, the vast majority of alpha l-noncanonical amino acids are accepted. Finally, the newly synthesized protein is released from the ribosome and folded, processed, and trafficked to its appropriate location. Since GCE incorporates Ox-PTMs into the primary sequence, altered protein folding pathways and cofactor loading are possible from the modified protein.

Alternative methods to GCE have been developed that may be applicable for particularly metabolically unstable or toxic ncAAs or toxic proteins. Expressed protein ligation (EPL) has emerged as another powerful method to study Ox-PTMs [33]. This method allows the vast chemical space open to solid phase peptide synthesis (SPPS) to be coupled with the robustness of recombinant protein expression. As the ncAA is incorporated via SPPS and then native chemical ligation, novel aaRS/tRNA pairs do not need to be generated. In addition, provided that the sites of modification are within <30 amino acids from one another, it is trivial to incorporate multiple ncAAs. Successful generation of modified histones with EPL also highlights some of the drawbacks of EPL, the site of interest should be within ~50 residues of the N- or C-terminus or a synthesis with three peptides is required, the protein of interest should be able to be refolded from denaturants, and there is some level of sequence requirement both for the intein to generate the *α*-thioester and for the presence of a cysteine at the site of ligation [34, 35]. The EPL strategy has been used to yield milligram quantities of *α*-synuclein nitrated selectively at Y39 or Y125 allowing biophysical and biochemical studies of site-specific nitration on *α*-synuclein structure and function [36]. It is also important to note that the standard desulfurization reaction conditions originally used reduced the incorporated nitrotyrosine to aminotyrosine. This reduction during the desulfurization reaction was prevented with the addition of 2-nitrobenzylamine hydrochloride.

## 5. Oxidative Modifications of Sulfur Containing Residues

Cysteine Ox-PTMs are abundant modifications with the cysteine sulfur existing in several different oxidation states. Cysteine sulfenic acid (Cys-SOH, 1) is directly generated by the oxidation of cysteine by two-electron oxidants, particularly H_2_O_2_. The propensity of Cys residues to undergo oxidation is influenced generally by the thiol nucleophilicity, the surrounding protein microenvironment, and the proximity of the target thiol to the ROS source [37]. Accordingly, the susceptibility to oxidation is usually correlated with the Cys pKa. Further, increasing evidence shows that ROS signaling responses are compartmentalized and that spatial regulation of Cys oxidation is key for signaling [38, 39]. Cys-SOH can be overoxidized to cysteine sulfinic acid (Cys-SO_2_H, 2). As the H_2_O_2_-mediated pathway of Cys-SOH oxidation proceeds through the sulfenate anion (Cys-SO^−^), the pKa of Cys-SOH should influence this reaction [39]. With a pKa of ~2, Cys-SO_2_H exists exclusively in a deprotonated state at physiological pH. The sulfinate group is usually not reducible by typical cellular reductants and as such its further oxidation to sulfonic acid appears to be the only relevant reaction in cells [40]. All of which points to the importance of temporal and spatial control of these protein modifications and the need for tools that enable further investigation.

Cys-SOH has been identified in a relatively small number of proteins, and the identification of this modification remains difficult. The first general analysis of known Cys-SOH modification sites included 47 proteins characterized by crystallography to contain the modification [41]. On the other hand, Cys-SO_2_H was long considered merely an artifact of protein purification. Increasing evidence however indicates that hyperoxidation to Cys-SO_2_H in cells is not a rare event. In fact, quantitative analysis of rat liver proteins has shown that ~5% of Cys residues exist as Cys-SO_2_H [42]. The discovery of sulfiredoxin (Srx), an enzyme that in an ATP-dependent protein reduces Cys-SO_2_H to Cys-SOH on some peroxiredoxins, has indicated that Cys-SO_2_H plays a biological role in the redox regulation of peroxiredoxin function [43].

The same electrostatic interactions on the protein that affect the pKa of the Cys thiol also influence the stability of the Cys-SOH. The major factor that increases Cys-SOH stability (or lifetime on a protein) is the absence of proximal thiols capable of generating an intramolecular disulfide (8). It has been also reported that limited solvent access and nearby H-bond acceptors also contribute to Cys-SOH stabilization. In addition to the reaction of Cys-SOH with neighboring cysteine thiols, backbone amide nitrogens can readily react with Cys-SOH to yield a cyclic sulfonamide species (5) [44, 45]. If Cys-SOH modifications are not removed by neighboring Cys residues or amide nitrogens, they can be enzymatically removed. Thioredoxin can directly reduce Cys-SOH to Cys-SH, and Cys-SOH reacts with glutathione to form a mixed disulfide (6), which is later reduced by glutaredoxin. Based on the rate of formation and repair by these mechanisms, the cellular lifetime of sulfenic acid is on the order of minutes, consistent with the lifetime of many PTMs, including phosphorylation [37]. In A431 cells, a peak of protein sulfenylation was observed five minutes after endothelial growth factor stimulation, with a subsequent decay over 30 minutes [39]. Due to the low stability and high turnover rates, monitoring their formation is problematic by direct mass analysis of Cys-SOH. Currently, the use of chemical probes is the only suitable technique to monitor Cys-SOH formation [46]. The low biological stability of Cys-SOH presents a significant challenge to GCE; however, chemical caging strategies and structural mimetics have been used to overcome this challenge [27, 30, 31]. Since Cys-SO_2_H is notably more stable, there is a good chance that this oxidation state can be directly incorporated via GCE, although modulation of Srx proteins may be necessary.

The biological impact of protein Cys-SOH formation has been particularly well outlined in protein tyrosine phosphoregulation. Cysteine oxidation controls the activity of both protein tyrosine kinases (PTKs) as well as protein tyrosine phosphatases (PTPs). Sulfenylation of the PTP catalytic Cys residue (pKa 4–6) has emerged as a dynamic mechanism for the inactivation of this protein family [47]. In comparison to PTPs which are always inactivated by ROS, oxidation of PTKs can result in either enhancement or inhibition of kinase activity [48, 49]. It is well established that ROS play a regulatory role for some ion channels, although little is described in terms of the molecular basis for this regulation [50]. Peroxiredoxins (Prxs) are important mediators of H_2_O_2_ signaling as they both maintain low levels of H_2_O_2_ and are themselves modified to Cys-SOH and Cys-SO_2_H to modulate H_2_O_2_ levels [51].

Cysteine is also S-nitrosylated following the production of NO (4), with implications regarding the influence of NO in cellular transduction [52]. Proteins with a wide variety of functions are found to be endogenously S-nitrosylated in intact cellular systems [53]. Much like other Ox-PTMs, it has become clear that S-nitrosylation and de-nitrosylation are regulated spatially and temporally in the cell [54]. Using the biotin switch methodology (or variations thereof), multiple proteins with the Cys-SNO modification have been isolated [55]. Among the identified proteins is GAPDH, which transnitrosylates and alters the enzymatic activity of SIRT1 [56]. Effector mechanisms for S-nitrosylation include protein-protein interactions, subcellular localization of proteins, and ubiquitylation-dependent protein degradation, which underlie a variety of cellular processes including apoptosis, metabolism, and membrane trafficking. This modification has been implicated in pathophysiological conditions including multiple sclerosis, Parkinson's disease, and asthma [57, 58]. As this Ox-PTM is unstable, genetic incorporation will likely require generation of a structural analogue similar to the methods employed for incorporation of phosphotyrosine and phosphoserine [30, 31]. Clearly, both cysteine oxidation and S-nitrosylation-based Ox-PTMs are of significant biological interest and this is a field ripe for the development of GCE tools.

Methionine sidechains also contain a sulfur atom susceptible to oxidation. ROS and reactive chlorine species are a major source of methionine oxidation yielding methionine sulfoxide (Met-SO, 11) [59, 60]. Methionine is a strongly hydrophobic residue and is generally buried, which protects it from oxidation, although those few surface exposed Met residues are susceptible to oxidation. Methionine oxidation yields two stereoisomers of the sulfoxide, S and R forms. Met-SO formation results in a much more hydrophilic amino acid than Met, which may affect protein structure. Although Met-SO is a fairly stable product, the sulfur can be further oxidized by strong oxidants to the sulfone (Met-SO_2_, 12); however, this occurs to a low extent [9]. Met-SO_2_ is considered an irreversible reaction product and cannot be converted back to Met by cellular reductants. In much the same way as Cys Ox-PTMS, tools to study Met Ox-PTMs are necessary in order to further explore the implications of these modifications.

Under conditions of H_2_O_2_ treatment in which Jurkat T-cells were 90% viable, more than 2000 oxidation-sensitive Met residues were identified in the proteome. The majority (84%) of Met-containing peptides contained a low degree of Met-SO (less than 30% oxidized), while only the remaining 16% of peptides were oxidized to a high degree (up to 100% Met-SO) [61]. This significant level of Met oxidation in biological systems requires robust enzymatic repair mechanisms. Methionine sulfoxide reductases (Msrs) efficiently repair Met-SO to Met and are present in all aerobically respiring organisms [62]. Met-SO reductases A and B (MsrA and MsrB) are the prototypical Msrs for the two Met-SO epimers, and while they are similar in neither sequence nor structure, they do share common mechanisms to reduce Met-SO to Met [63].

Methionine oxidation is associated with the aging process and several pathophysiological conditions such as neurodegenerative diseases and cancer [64, 65]. Previously, Met-SO formation under these conditions was regarded only as protein damage. However, Met oxidation is now being acknowledged as a mode of triggering protein activity. The kinase CaMKII and the transcription factor HypT were both found to be activated following oxidation of particular methionines [66, 67]. The polymerization of actin has also been shown to be regulated by the redox state of Met residues, mediated by the concerted and stereo-selective action of Mical proteins and MsrB1 [68].

Oxidized cysteine or methionine residues have yet to be incorporated by GCE. While this should not be an insurmountable challenge, sulfur containing Ox-PTMs do present stability issues. Cys-SOH is not stable on proteins in living cells, so genetic incorporation will require a chemical caging strategy or the use of a mimetic, analogous to what has been done for stable mimetics of phosphorylated serine, threonine, and tyrosine [27, 29–31]. A photocaged Cys-SOH on the protein Gpx3 has been prepared by alkylation of catalytic Cys32 with dimethoxy-*o*-nitrobenzyl bromide (DMNB-Br), followed by oxidation with H_2_O_2_. While the photocaged cysteine sulfenic acid free amino acid was also synthesized with the goal to genetically encode this amino acid, to date, it has not been incorporated via GCE [69]. In order to successfully incorporate Met-SO, modulation of cellular Msr levels will be imperative in order to purify intact modified protein similar to the hurdles of removing cellular phosphatases when incorporating phosphorylated amino acids [31].

## 6. Ox-PTMs of Aromatic Residues

While the role of sulfur oxidation has been extensively studied, the biological role of Ox-PTMs on aromatic residues is less clear. Residues susceptible to oxidative or nitrosative modifications include tyrosine, tryptophan, and histidine.

Protein tyrosine nitration (nitroTyr, 13) occurs under basal physiological conditions and is several-fold enhanced under conditions of increased oxidant and ^•^NO formation. Much like other Ox-PTMs, the distribution of tyrosine-nitrated proteins is largely dependent on the proximity to sites of RNS generation [70]. With the advent of proteomic analyses, it has been observed that protein tyrosine nitration occurs on a subset of proteins, and within those proteins, only a subset of tyrosines is nitrated [13, 71–74]. Based on current evidence, the mechanism of protein tyrosine nitration in biological systems is mediated by free radical reactions, implying an intermediate tyrosyl radical and subsequent reactions with either ^•^NO or ^•^NO_2_ [75]. Other Ox-PTMs may result from this general reaction mechanism as tyrosyl radical may also react with ROS to form l-3,4-dihydroxyphenylalanine (DOPA, 14) or another nearby tyrosine to form the protein-cross-linked dityrosine (17) [76, 77]. Tyrosine is also susceptible to modification by myeloperoxidase- and eosinophil peroxidase-derived hypochlorous and hypobromous acid to form 3-chlorotyrosine (21) and 3-bromotyrosine (23) [13]. As with all Ox-PTMs, it is difficult to establish direct and quantitative relationships between extent of nitration on specific proteins and biological responses in cells and the influence of protein tyrosine nitration is often obscured by the multiplicity of oxidative modifications. GCE has already begun to untangle some of this complexity [78–81].

Mass spectrometry indicates that protein-bound nitroTyr is present in plasma and tissue at levels on the order of 1 *μ*mol of nitroTyr/mol of tyrosine under normal conditions and increases up to 100-fold under conditions of oxidative stress [82, 83]. Over 60 individual proteins have been determined to contain nitroTyr [10] of which several have been investigated further with GCE. Less is known about the abundance of other tyrosine Ox-PTMs although in general they appear to be less abundant and appear on fewer proteins [13].

In contrast to the previously discussed Ox-PTMs, nitroTyr and the other tyrosine Ox-PTMs are generally stable modifications requiring protein turnover to remove the Ox-PTM-modified proteins from the cellular protein pool [84]. A “denitrase” activity, capable of returning nitroTyr to the native tyrosine, has been reported multiple times although the enzyme responsible has not been isolated [85–88]. Tyrosine nitration is abundant in aging tissue and has been linked to pathological conditions including neurodegeneration, atherosclerosis, and cancer [10, 89]. While tyrosine nitration was traditionally thought of as global oxidative damage that accumulates under conditions of oxidative stress, it has become clear that some proteins with nitroTyr modifications at specific sites are capable of mediating biology [79–81]. Currently, it is unclear the structural consequences of adding a meta nitro group to a tyrosine on the proteins that have this clear gain of function. The most obvious two options for altering protein structure are from the pKa change to tyrosine and new interactions afforded by the nitro group. Nitration of tyrosine lowers the pKa of the amino acid from ~10 to near neutral pH, imparting a negative charge, while the nitro group also adds significant steric bulk and new hydrogen bonding groups. GCE is uniquely positioned to determine the structural consequences of PTMs because structural mimics of a PTM can be installed to probe if one chemical feature of a PTM is more critical than another. Possible biological effects due to these alterations to tyrosine properties include changes in protein activity (gain- or loss-of-function), increased protein immunogenicity, interference in tyrosine kinase-dependent regulation, modulation of protein assembly or polymerization, facilitation of protein degradation (turnover), or formation of proteasome resistant protein aggregates [90]. GCE has provided tools to further the molecular understanding of protein tyrosine nitration, and development of new tools promises further control over the study of biological processes associated with this Ox-PTM.

High levels of protein tyrosine halogenation have been detected in several inflammatory conditions including arthritis, some types of cancer, heart disease, cystic fibrosis, and asthma [91–93]. Protein tyrosine halogenation *in vivo* is a result of the reaction of myeloperoxidase-derived HOCl or eosinophil peroxidase-derived HOBr, yielding 3-chlorotyrosine (21) and 3-bromotyrosine, respectively (23). HOCl and HOBr are strong oxidants and possess potent antibacterial properties, as such they are generated as components of mammalian host defense [94, 95]. However, overproduction or misplaced induction can lead to accumulation of protein modification seen in the above inflammatory conditions. The 3-chlorotyrosine modification along with 3-nitroTyr has been noted in ApoA1 [91, 96]. The interest in these Ox-PTMs has led to the generation of a PylRS-pylT pair that efficiently incorporates both 3-chlorotyrosine and 3-bromotyrosine.

The same conditions that result in protein tyrosine nitration also result in oxidation and nitration of tryptophan residues. A variety of products result from the *in vitro* reaction of tryptophan with biologically relevant RNS (27–44), but those hydroxytryptophan and nitrotryptophan have been detected in samples from tissue or cell culture, specifically 2-hydroxytryptophan (27), 4-hydroxytryptophan (28), 5-hydroxytryptophan (29), or 6-hydroxytryptophan (30), 6-nitrotryptophan (36), and 5-hydroxy-6-nitrotryptophan (40) [7, 13, 97].

While tyrosine and tryptophan Ox-PTMs occur under the same conditions, several MS studies have indicated that tryptophan modifications are on the order of 10- to 1000-fold less abundant than tyrosine modification [98, 99]. No direct repair mechanisms for tryptophan Ox-PTMs have been noted, indicating that the only path to remove these Ox-PTMs is protein turnover. Although Ox-PTMs occur at a lower rate on tryptophan than on tyrosine, these modifications likely modulate cellular regulation as tryptophan residues are particularly important for specific protein-protein interactions and protein-small molecule recognition [100, 101]. At least one instance of 5-hydroxy-6-nitrotryptophan formation has been reported in the mitochondrial metabolic enzyme succinyl-CoA:3-ketoacid coenzyme A transferase (SCOT). This age-dependent Ox-PTM leads to a 30% increase in SCOT enzymatic activity and is thought to play a protective role, allowing the heart to better utilize ketone metabolism for energy production [102].

While a novel GCE system for incorporation of tryptophan derivatives has been published and shown to utilize 5-hydroxytryptophan as a substrate, it has not yet been utilized to investigate the effects of this Ox-PTM [22]. As tryptophan Ox-PTMs are generally stable to cellular conditions and the tryptophan-based system can be evolved for other tryptophan-based ncAAs, this system will likely be applicable to the study of tryptophan Ox-PTMs.

Oxidation of histidine to 2-oxohistidine (45) occurs through a radical mechanism in protein, particularly near metal binding sites or in tissue exposed to UV radiation [103]. This Ox-PTM has been noted to occur significantly in Cu, Zn-SOD [104]. Little is known about how widespread this modification is or how it impacts biological function [105]. While a variety of histidine mimics have been genetically encoded via GCE, 2-oxohistidine has not yet been encoded genetically [106]. As 2-oxohistidine is stable enough on proteins to be characterized by X-ray crystallography [107] and given the aforementioned orthogonal system for modified histidine incorporation, it is likely that 2-oxohistidine will be amenable to incorporation by GCE.

## 7. Ox-PTMs that Have Been Incorporated by GCE

The first PTM genetically encoded was nitroTyr by Neumann and coworkers in 2008 and to date has been the most utilized GCE system for probing Ox-PTMs. An *Mj* orthogonal pair was evolved and subsequently used to confirm a loss of function in site-specifically nitrated MnSOD [78]. Through a novel selection scheme, a second generation *Mj* nitroTyr-RS was then generated with roughly an order of magnitude greater efficiency for producing nitroTyr-proteins [23]. This second generation *Mj* nitroTyr-RS was used to generate all 5 forms of nitrated Hsp90 found in endogenously nitrated Hsp90. This allowed each different site-specifically nitrated form of Hsp90 to be characterized for functional changes *in vitro* and cellular toxicity *in vivo* in different mammalian cell lines. Three of the five nitrated forms had no apparent effect when delivered into PC12 cells at physiologically relevant levels. For nitrated Hsp90, a toxic gain of function and motor neuron cell death occur if Hsp90 is nitrated at either tyrosine 33 or tyrosine 56 in less than 5% of the cellular pool of Hsp90 [79]. This is a key advance in the study of Ox-PTMs because it is the first example of a toxic gain of function confirmed from a single site of modification on a protein. Antibodies to nitrated Hsp90 were then generated, and using GCE was validated to recognize site-specific nitroTyr-33-Hsp90 and nitroTyr-56-Hsp90. These antibodies, now confirmed to recognize the nitration of a specific sites on Hsp90, have been used to monitor formation of toxic nitrated Hsp90 in tissue and track these oxoforms in other pathological states. It was further shown that nitration of Hsp90 at tyrosine 33 downregulates mitochondrial activity [81]. This nitroTyr GCE system has also been used to investigate physiological role of tyrosine nitration in apolipoprotein A1 (ApoA1), a serum protein that facilitates systemic lipid trafficking. Using mass spectroscopy, three tyrosine residues in ApoA1 were identified as nitration sites. Genetic code expansion allowed the generation and characterization of each nitrated form of ApoA1, and only one nitrated tyrosine had an effect on ApoA1 activity. GCE was used to show that site-specific nitration of ApoA1 at tyrosine 166 results in a 90% reduction in LCAT activity [80]. A site-specific antibody for ApoA1 nitroTyr-166 was also generated in order to monitor this Ox-PTM in the context of atherosclerotic tissue. These antibodies specific for ApoA1 nitroTyr-166 confirmed that only this nitrated variant was removed from human serum and was enriched in atherosclerotic plaques by 1000-fold over nonnitrated ApoA1.

The ability to incorporate the structurally similar 3-chlorotyrosine and 3-bromotyrosine Ox-PTMs with GCE would enable the characterization of these modifications in disease. However, the first and second generation *Mj* nitroTyr synthetase is not permissive to these ncAAs, and synthetases generated for halotyrosine using the *Mj* synthetase system resulted in poor efficiency and fidelity. However, selections for a 3-chlorotyrosine synthetase from the PylRS/pylT pair have yielded aaRSs that have good efficiency and fidelity for halogenated tyrosines. Proteins with site-specifically incorporated 3-chlorotyrosine and 3-bromotyrosine have indicated that currently available commercial antibodies to these modifications are not specific or are not sensitive to these modifications alone. GCE now presents the opportunity to improve on the specificity and sensitivity of antibodies for these modifications.

GCE has also been applied to the site-specific incorporation of the redox-active amino acid DOPA (14). The site-specific incorporation of DOPA is challenging because when this amino acid is added to media, it can react with cellular components, yielding the oxidized ncAA (DOPA quinone, 15) that can serve as an electrophile [108]. To overcome the side reactions in media, a photocaged DOPA has also been encoded [109]. While the GCE of photocaged DOPA was developed for the production of recombinant bioadhesives not for studying oxidative stress, this tool could also be used to evaluate the effects of DOPA containing proteins in disease.

## 8. Application of GCE to the Study of Ox-PTMs

**Figure 4:**
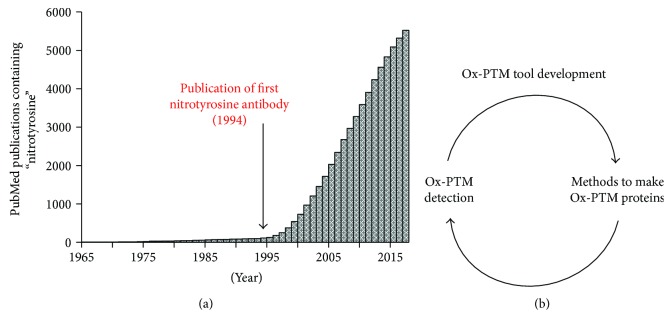
(a) Total number of publications on PubMed containing the word ‘nitrotyrosine' in the title or abstract published up to the year indicated. The publication of the first nitrotyrosine antibody by Beckman and coworkers in 1994 is denoted with an arrow. (b) The cycle of development of chemical biology tools for studying Ox-PTMs. New methods like development of antibodies allow easier detection of Ox-PTMs, which naturally lead to increased interest and development of new chemical biology tools, including GCE, for studying Ox-PTMs. Tools like GCE allow for development of better detection methods, which further reinforces the cycle.

It has long been known that ROS and RNS modify protein amino acids but it has been challenging to determine the extent of their biological role and abundance. Identifying Ox-PTM formation on a protein is the first step of any investigation into Ox-PTMs followed by verification of biological relevance. The nitroTyr Ox-PTM can be used as an example of how the synergy of new detection methods and GCE can advance the understanding of Ox-PTMs. The earliest manuscripts outlining detection of nitroTyr in proteins were based on methods using HPLC/UV-Vis and amino acid analysis because this Ox-PTM possesses a significant absorbance at 430 nm [110, 111]. A September 2017 PubMed literature survey revealed 5522 manuscripts published with the term “nitrotyrosine” in the title or abstract. In the first 24 years of research involving nitroTyr, less than 2% of present publications on nitroTyr were published, which was followed by a virtual explosion in the number of papers published on nitroTyr in the ensuing years. This rapid increase in nitroTyr research coincides nicely with the development of the first nitroTyr antibodies by Joe Beckman and coworkers in 1994 (Figure 4(a)) [112]. NitroTyr antibodies have been used to detect this modification in tissue via immunohistochemistry, for identification of proteins via Western blot and for enrichment of proteins for proteomic methods. Due in part to access to antibodies as a detection method, nitroTyr now serves as a general biomarker for oxidative stress [113].

The increased interest in the nitroTyr Ox-PTM has spurred numerous methods for identifying nitroTyr proteins including HPLC-based techniques for quantifying total nitroTyr in tissues and lysates and various mass spectrometry methods for identifying sites of nitroTyr modification in proteins [11, 114]. The interest in nitroTyr and its detection in a variety of pathological conditions created the need to generate homogeneous nitrated proteins for characterization which led directly to the first GCE system for nitroTyr incorporation [78]. GCE clearly enables characterization of how a specific Ox-PTM on a protein alters its function but it also allows for analysis of the antibodies generated to detect Ox-PTM proteins. With access to site-specific and homogeneous Ox-PTM protein generated from GCE, the specificity and sensitivity of Ox-PTM antibodies can be determined. The original nitroTyr antibodies generated were a foundational advancement in studying oxidative stress, but as the field progresses, improved antibodies for studying Ox-PTMs are needed. While the development of nitroTyr antibodies lead to the incorporation of nitroTyr via GCE, better detection methods for nitroTyr can now be developed using site-specifically modified protein from GCE (Figure 4(b)).

It has become very clear through GCE that nitroTyr antibodies have wildly different sensitivity depending on the site of protein nitration. All of the currently available Ox-PTM antibodies were developed prior to GCE of Ox-PTM and were validated using methods available at the time of their development [115]. Now, GCE allows for verification that Ox-PTM antibodies are selective for one type of Ox-PTM over others of similar structure. Since antibodies are often generated to proteins that have been exposed to ROS and RNS reagents, not homogeneous Ox-PTM proteins, the resulting antibody specificity might be to a different Ox-PTM than intended. For example, the nitroTyr monoclonal antibody (clone 1A6) indicated a nitrated protein present in aged rat heart mitochondria, but this protein was instead found to contain 5-hydroxy-6-nitrotryptophan, which closely resembles the nitroTyr sidechain [102].

A powerful application of GCE is the ability to confirm that an Ox-PTM modification at a specific location in a protein is detected by an antibody. While nitroTyr-antibodies are specific for the nitroTyr modification, they do not detect all sites of nitration equally. In addition, if there is the ability to detect the modification at one location on a protein and not another, this can be verified with GCE. A specific peptide sequence containing the desired Ox-PTM can be used to generate antibodies, and then the Ox-PTM antibodies can be screened for selectivity against homogeneous protein generated with GCE. Antibodies specific for nitroTyr-33-Hsp90, nitroTyr-56-Hsp90, and ApoA1 have been generated using this approach [79–81]. These site-specific antibodies have been used to determine the extent of nitration of specific sites in Hsp90 in different cellular contexts and conditions. Unsurprisingly, there exists clear peptide context-dependent sensitivity to the function of nitroTyr antibodies, that is to say, the amino acid sequence of the protein surrounding the Ox-PTM site plays a role in antibody detection sensitivity. In addition to the development of site-specific antibodies, the ability of GCE to produce homogeneously nitrated proteins allows for the characterization of this context dependence that was not possible with existing techniques. While much of the past work on detection of Ox-PTMs with antibodies has focused on nitroTyr, further development of GCE for other Ox-PTMs will lead to exciting advances in Ox-PTM antibody development and validation, particularly for cysteine and methionine oxoforms.

As Ox-PTMs are relevant to disease, the Ox-PTM proteins and their function represent a new class of possible therapeutic targets. Any Ox-PTM protein that shows an undesirable gain-of-function or new interaction could be a therapeutic target. Ox-PTMs present a unique situation insofar as they are not directly enzymatically catalyzed and therefore the PTM “writer” (e.g., kinase) cannot be directly inhibited. This requires the oxidized protein itself to be directly inhibited. For instance, nitrated Hsp90 found in motor neurons under pathological conditions such as ALS and spinal cord injury may present a target for intervention [79]. It has become increasingly clear that the site of protein Ox-PTMs is an important determinant of their biological role. With this in mind, the use of GCE for site-specific PTM incorporation as a means of screening for potential therapeutics against specific Ox-PTMs has been acknowledged [116]. This is clear in the case of Hsp90, which is endogenously nitrated on five tyrosines and of them only nitration of tyrosines 33 and 56 leads to motor neuron cell death, while nitration of tyrosine 33 downregulates mitochondrial activity [79, 81]. Given this clear functional specificity from site-dependence of nitrated Hsp90, it will be desirable to develop sequence-specific inhibitors that target site-specific Ox-PTM proteins. As GCE can produce all possible Ox-PTM forms, the technology will allow for screening of therapeutic compounds against site-specific oxoforms.

## 9. Conclusions and Perspective

The study of protein Ox-PTMs is hampered by access to defined Ox-PTM proteins. GCE is uniquely suited to overcome this roadblock because it generates site-specific and homogenous Ox-PTM proteins. GCE has been applied to protein tyrosine nitration both to investigate biological effects of particular sites of modification on key proteins and to provide defined Ox-PTM proteins for validating nitroTyr antibodies. Every newly identified Ox-PTM protein that shows a gain of function can be considered as a therapeutic target since it forms under oxidative stress conditions. As seen with nitrated Hsp90 and ApoA1, the ability to generate Ox-PTM proteins opens the ability to develop screens for therapeutics and identifies their molecular role in disease.

Thus far, the development of GCE methods for Ox-PTMs has relied on researchers from both the oxidative stress field and the GCE fields. NitroTyr was identified in biological samples as a major marker for oxidative stress which prompted those in GCE to develop methods for site-specific incorporation of this Ox-PTM. As those in the oxidative stress field embraced the use of the GCE methods for challenging problems, the GCE tools required refinement and improvement. With this in mind, significant advances in the Ox-PTM field will likely rely on synergy between developments in GCE technology and those applying the tools to challenging Ox-PTM problems.

## Supplementary Materials

Supplementary Material Table S1: list of Ox-PTMs identified following oxidative stress in cells or exposure of protein *in vitro* to ROS and RNOS.
