# Supplementary material for: Genetic Code Expansion: A Powerful Tool for Understanding the Physiological Consequences of Oxidative Stress Protein Modifications
Source: Oxid Med Cell Longev. 2018 Apr 23;2018:7607463. doi: 10.1155/2018/7607463 (PMC5937447; doi:10.1155/2018/7607463)
Supplement: Supplementary Material — Table S1: list of Ox-PTMs identified following oxidative stress in cells or exposure of protein in vitro to ROS and RNOS. [file 7607463.f1.pdf]

**SUPPLEMENTARY TABLE S1**

| Number | Ox-PTM Name | Reference |
|---|---|---|
| (**1**) | cysteine sulfenic acid | [2, 3] |
| (**2**) | cysteine sulfinic acid | [2, 3] |
| (**3**) | cysteine mixed disulfide | [4] |
| (**4**) | S-nitrosyl cysteine | [3] |
| (**5**) | cysteine cyclic sulfenamide | [2, 3] |
| (**6**) | cysteine glutathionylation | [3] |
| (**7**) | cysteine thiosulfinate | [2] |
| (**8**) | cysteine disulfide | [2-4] |
| (**9**) | cysteine sulfonic acid | [2, 3] |
| (**10**) | Prx cysteine phosphoryl sulfinic enzyme intermediate | [2] |
| (**11**) | methionine sulfoxide | [4, 5] |
| (**12**) | methionine sulfone | [5] |
| (**13**) | 3-nitrotyrosine | [6] |
| (**14**) | 3,4-dihydrophenylalanine (DOPA) | [6] |
| (**15**) | dopaquinone (DQ) | [6] |
| (**16**) | DQ protein conjugate adduct | [6] |
| (**17**) | 3,3'-dityrosine | [6] |
| (**18**) | 3-aminotyrosine | [6] |
| (**19**) | Quinone imine (QI) | [6] |
| (**20**) | QI protein conjugate adduct | [6] |
| (**21**) | 3-chlorotyrosine | [6] |
| (**22**) | 3,5-dichlorotyrosine | [6] |
| (**23**) | 3-bromotyrosine | [6] |
| (**24**) | 3,5-dibromotyrosine | [6] |
| (**25**) | 3-iodotyrosine | [6] |
| (**26**) | 3,5-iodotyrosine | [6] |
| (**27**) | 2-hydroxytryptophan | [4, 7] |
| (**28**) | 4-hydroxytryptophan | [4, 7] |
| (**29**) | 5-hydroxytryptophan | [4, 7] |
| (**30**) | 6-hydroxytryptophan | [4, 7] |
| (**31**) | 7-hydroxytryptophan | [4, 7] |
| (**32**) | dihydroxytryptophan | [7] |
| (**33**) | 2-nitrotryptophan | [7] |
| (**34**) | 4-nitrotryptophan | [7] |
| (**35**) | 5-nitrotryptophan | [7] |
| (**36**) | 6-nitrotryptophan | [7] |
| (**37**) | 7-nitrotryptophan | [7] |

| Number | Ox-PTM Name | Reference |
|---|---|---|
| (**38**) | 1-nitrotryptophan | [7] |
| (**39**) | 1-nitrosotryptophan | [7] |
| (**40**) | 5-hydroxy-6-nitrotryptophan | [7] |
| (**41**) | N-formylkynurenine | [4, 7] |
| (**42**) | 3-hydroxykynurenine | [4] |
| (**43**) | Oxindole-3-alanine | [7] |
| (**44**) | kynurenine | [7] |
| (**45**) | 2-oxohistidine | [4, 8] |
| (**46**) | Asparagine (Ox-PTM histidine) | [4] |
| (**47**) | Aspartate (Ox-PTM histidine) | [4] |
| (**48**) | 4-hydroxyphenylalanine | [4] |
| (**49**) | 2-hydroxyphenylalanine | [4, 6] |
| (**50**) | 3-hydroxyphenylalanine | [4, 6] |
| (**51**) | 2-alkenal Michael adduct (Ox-PTM histidine) | [8] |
| (**52**) | 4-hydroxy-2-alkenal Michael adduct (Ox-PTM histidine) | [8] |
| (**53**) | Aminadipic semialdehyde (Ox-PTM lysine) | [4, 8] |
| (**54**) | 2-alkenal Michael adduct (Ox-PTM lysine) | [8] |
| (**55**) | 4-hydroxy-2-alkenal Michael adduct (Ox-PTM lysine) | [8] |
| (**56**) | FDP adduct (Ox-PTM lysine) | [8] |
| (**57**) | N-propenallysine | [8] |
| (**58**) | DHP adduct (Ox-PTM lysine) | [8] |
| (**59**) | pyridium DHP (Ox-PTM lysine) | [8] |
| (**60**) | Glutamylsemialdehyde (Ox-PTM arginine) | [4] |
| (**61**) | 3-hydroxyleucine | [4] |
| (**62**) | 4-hydroxyleucine | [4] |
| (**63**) | 3-hydroxyvaline | [4] |
| (**64**) | 4-hydroxyglutamic acid | [4] |
| (**65**) | 2-amino-3-ketobutyric acid | [4] |

Supplementary Table S1: List of Ox-PTMs identified following oxidative stress in cells or exposure of protein *in vitro* to ROS and RNOS.
